# MASP-1 of the complement system enhances clot formation in a microvascular whole blood flow model

**DOI:** 10.1371/journal.pone.0191292

**Published:** 2018-01-11

**Authors:** Lorenz Jenny, József Dobó, Péter Gál, Gábor Pál, Wilbur A. Lam, Verena Schroeder

**Affiliations:** 1 Experimental Haemostasis Group, Department for BioMedical Research, University of Bern, Bern, Switzerland; 2 Institute of Enzymology, Biological Research Center, Hungarian Academy of Sciences, Budapest, Hungary; 3 Department of Biochemistry, Eötvös Loránd University, Budapest, Hungary; 4 Wallace H. Coulter Department of Biomedical Engineering, Georgia Institute of Technology and Emory University, Atlanta, Georgia, United States of America; University of Oxford, UNITED KINGDOM

## Abstract

The complement and coagulation systems closely interact with each other. These interactions are believed to contribute to the proinflammatory and prothrombotic environment involved in the development of thrombotic complications in many diseases. Complement MASP-1 (mannan-binding lectin-associated serine protease-1) activates coagulation factors and promotes clot formation. However, this was mainly shown in purified or plasma-based static systems. Here we describe the role of MASP-1 and complement activation in fibrin clot formation in a microvascular, whole blood flow model. This microfluidic system simulates blood flow through microvessels at physiological flow and shear rates and represents the closest model system to human physiology so far. It features parallel microchannels cultured with endothelial cells in a transparent microfluidic chip allowing real-time evaluation of clot formation by confocal microscopy. To test their effects on clot formation, we added the following activators or inhibitors (individually or in combination) to whole blood and performed perfusion experiments: rMASP-1cf (recombinant active form of MASP-1), complement activator zymosan, selective MASP-1 inhibitor SGMI-1 (based on the *Schistocerca gregaria* protease inhibitor scaffold), classical pathway inhibitor rSALO (recombinant salivary anti-complement from *Lutzomyia longipalpis*). Addition of rMASP-1cf resulted in accelerated fibrin clot formation while addition of SGMI-1 delayed it. Complement activation by zymosan led to increased clot formation and this effect was partially reversed by addition of rSALO and almost abolished in combination with SGMI-1. We show for the first time a strong influence of MASP-1, complement activation and pathway-specific inhibition on coagulation in a microvascular flow system that is closest to human physiology, further underpinning the *in vivo* relevance of coagulation and complement interactions.

## Introduction

Cardio- and cerebrovascular diseases (CVDs) still represent the leading cause of morbidity and mortality in industrialized countries, despite the availability of modern therapies. The development of CVDs is a consequence of a proinflammatory and prothrombotic vascular environment that may be further promoted by dysregulation of the complement system and its interactions with the coagulation system. Therefore, targeting the complement system and its interactions with coagulation may in the future represent a promising novel approach in the prevention and therapy of CVDs.

The complement and coagulation systems share a common evolutionary origin, they show many similarities, and there is increasing evidence for their close interaction in order to protect the body in case of injury and infection. The extensive cross-talk between complement and coagulation is reciprocal and occurs on all levels of their cascades, and therefore needs tight regulation. A dysregulation of one or the other cascade may lead to an excessive activation of both systems, which can become manifest in many diseases including infection, sepsis, diabetes and atherosclerosis [1–3].

The complement system is an essential part of the innate immune system and serves to eliminate pathogens from the circulation, it mediates the inflammatory response and is involved in the clearance of apoptotic host cells. The lectin pathway (LP) is one of three activation pathways, all of which lead to a common downstream pathway that triggers the three main effector functions of the complement system: i) Enhancing the inflammatory process by anaphylatoxins, ii) formation of the membrane attack complex (MAC, C5b-9) on cell surfaces, and iii) opsonizing surfaces, thereby marking them for clearance [4].

The LP is activated by binding of specific structures on microorganisms and altered self-surfaces to mannose-binding lectin (MBL) and other collectins or ficolins which are complexed in a diverse manner with the MBL-associated serine proteases (MASPs) MASP-1, MASP-2 or MASP-3. Upon binding of a target, MASP-1 becomes activated and changes its conformation, subsequently leading to an inter- and intra-complex activation of MASP-2 and additional MASP-1 [5]. Both activated MASP-1 and MASP-2 promote the formation of the C3-convertase via C2 and C4 cleavage and thereby trigger the effector functions of the complement system [4,6].

In recent years various interactions between the lectin pathway (LP) of complement and the coagulation system have been demonstrated. Among the LP components, especially MASP-1 has moved into the focus of interest: It has been shown that MASP-1 is more closely related to thrombin than to other complement serine proteases in terms of its structural features and its broad substrate specificity [7,8]. Besides its substrates in the LP, MASP-1 is also able to cleave thrombin substrates such as fibrinogen, blood coagulation factor XIII (FXIII), thrombin-activatable fibrinolysis inhibitor (TAFI), and protease-activated receptor 1 (PAR-1) on endothelial cells [9–11]. Furthermore, we have recently demonstrated that MASP-1 is able to induce clot formation in a prothrombin-dependent manner in thrombelastographic experiments and have presented the first model of MASP-1-mediated prothrombin activation [12,13]. MASP-1 has also been shown to be activated by platelets and fibrin formation in a prothrombotic environment [14] and suggested to modulate clot structure and resistance to fibrinolysis [9].

*In vivo* studies using animal models have provided strong evidence for an involvement of MASP-1 in coagulation. Takahashi et al. showed that MBL and MASP-1 knockout mice exhibited a prolonged bleeding time upon tail tip excision [15], while another study demonstrated that mice deficient in MBL and MASP-1 show decreased thrombus formation in FeCl_3_-induced thrombogenesis [16]. Furthermore, we have shown that MASP-1 and MASP-2 plasma levels are altered in patients with CVD [17].

So far, experiments studying the direct and indirect interactions of MASP-1 with the human coagulation system have mainly been conducted in purified or plasma-based static systems [9,14,18]. Tsai et al. reported the development of a microfluidic model which accurately simulates microvascular blood vessels in a way that is very close to human physiology [19]. It allows real-time confocal microscopic observation of blood flow and clot formation in parallel channels (dimensions 50 μm x 100 μm) in a transparent silicone chip coated with a viable endothelial cell monolayer perfused with whole blood at physiological flow and shear rates (1–4 dyne/cm^2^). The four parallel channels allow simultaneous observation of several samples, e.g. with addition of different activators or inhibitors, from the same donor under the same experimental conditions making this model an ideal tool to observe differences in clot formation. The model can easily be adapted for different experimental settings [20]. It has been validated for studies on platelet mechanics, hematological diseases and interactions between coagulation and the immune system [19,21–23].

For the present study, we have established this model in our laboratory in close collaboration with the group of Prof Wilbur Lam and used it to investigate the relevance of MASP-1 and complement activation for whole blood clot formation under flow conditions for the first time. We also show for the first time that MASP-1 and pathway-specific complement inhibition reduces clot formation in this close-to-physiological environment.

## Materials and methods

### Recombinant MASP-1 and specific inhibitors

So far it has not been possible to express or purify reasonable amounts of pure and stable full-length MASP-1; therefore, we have used a truncated recombinant form of MASP-1 termed rMASP-1cf (recombinant MASP-1 catalytic fragment) for our experiments. rMASP-1cf comprises the catalytic active C-terminal domains CCP1-CCP2-SP while lacking the N-terminal domains CUB1-EGF-CUB2 [7]. In a recent work, we showed that rMASP-1cf and full-length MASP-1 have the same effects on clot formation [12]. In our experiments we used a final concentration of 10 μg/ml rMASP-1cf, which corresponds to the average concentration measured in sera of healthy adult donors [24].

SGMI-1 (*Schistocerca gregaria* protease inhibitor (SGPI)-based MASP inhibitor-1), MW 4076 Da, is a monospecific inhibitor for MASP-1 [25]. SGMI-1 has been shown to have no significant effect on blood coagulation in the standard coagulation assays thrombin time (TT), prothrombin time (PT), and activated partial thromboplastin time (APTT) [26].

rSALO (recombinant salivary anti-complement from *Lutzomyia longipalpis*), MW 10.8 kDa, inhibits the activation of the classical complement pathway (CP). The exact mode of action of the molecule is unknown so far, however it has been shown to inhibit the CP-dependent deposition of C4b, C3b, C5b and C9, while not interfering with the activities of the lectin pathway, the alternative pathway (AP) and the proteases of the hemostatic system. It is assumed that rSALO interferes with CP activation by either displacing C1r or C1s from the C1 complex or by inhibiting the C1r activity [27].

### The microvascular flow model

**Fig 1:**
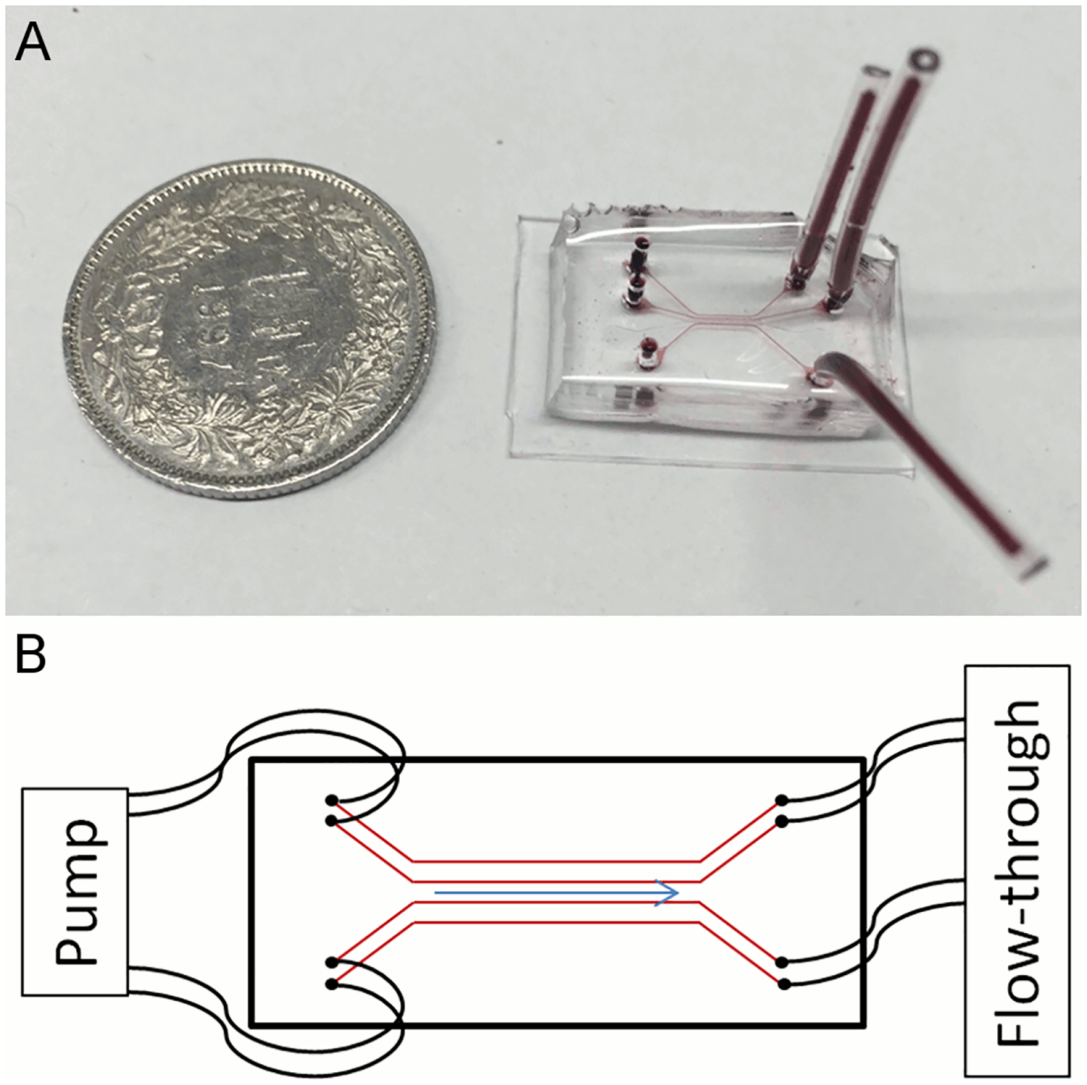
The microvascular flow model. (A) Polydimethylsiloxane chip (filled with a red dye for the purpose of making the channels visible) in size comparison with a Swiss one franc coin (diameter of 2.32 cm). (B) Cartoon of a chip with four parallel channels showing the tubings connecting pump and inlets, the outlets, and direction of flow.

The wafer serving as template for the chip was produced by imprinting a specific microchannel pattern onto a silicon photomask at the Georgia Institute of Technology and Emory University of School of Medicine in Atlanta, Georgia, USA. The single-use chips were then produced in our lab at the University of Bern: The wafer was covered with a solution of polydimethylsiloxane (PDMS) and its curing agent (Sylgard 184 silicone elastomer kit; Dow Corning, Midland, Michigan, USA) at a thickness of 1 cm and cured over-night at 60°C. Subsequently, holes of 1.42 mm diameter were punched into the chip connecting the parallel channels with the surface of the device. The bottom side bearing the microchannels (dimensions 50 μm x 100 μm) was subsequently sealed with a thin layer of PDMS (2 mm) by plasma bonding (basic plasma cleaner, Harrick Plasma, Ithaca, New York, USA). A finished chip and its schematic structure are shown in Fig 1.

The channels of the chip were coated with a solution of 5% fibronectin (final concentration 50 μg/ml, Sigma Aldrich, St. Louis, Missouri, USA) dissolved in PBS (pH 7.4, KH_2_PO_4_ 1.1 mM, NaCl 155 mM, Na_2_HPO_4_ 3 mM, without CaCl_2_ and MgCl_2_, Life Technologies, Carlsbad, USA) and incubated at 37°C, CO_2_ 5% for 60 min. In the meantime, passage 4–7 human umbilical vein endothelial cells (HUVECs; catalogue number CC-2519, obtained directly from Lonza in May 2015, Basel, Switzerland) were cultured to super-confluency (4.8x10^4^ cells/cm^2^) in endothelial cell growth medium (EGM-2, Lonza) in a T25 flask. The HUVECs were washed twice with PBS (pH 7.4, KH_2_PO_4_ 1.1 mM, NaCl 155 mM, Na_2_HPO_4_ 3 mM, without CaCl_2_ and MgCl_2_, Life Technologies) and trypsinized with 2 ml 0.05% trypsin in Hank’s balanced salt solution containing 0.53 mM EDTA (Dow Corning). Subsequently, the trypsin was inactivated with 9 ml EGM-2 and the cell suspension centrifuged at 1000 x g for 10 min. The pellet was resuspended in 80 μl EGM-2 containing dextran (80 mg/ml, 450–650 kDa; Sigma Aldrich) and applied to a Falcon tube with a cell strainer cap (35 μm mesh size; Dow Corning) to avoid cell clumping. The cells were then injected into the microchannels at a concentration of 1.2x10^6^ per ml. The chip was incubated at 37°C, 5% CO_2_ for 60 min before tubing (PTFE, inner diameter 0.012 inch, outer diameter 0.03 inch; Cole-Parmer, Vernon Hills, Illinois, USA) was connected to the channels and a constant EGM-2 flow of 2 μl/min was applied for 48 h. After two days, the channels were checked by microscopy to determine if a viable endothelial cell monolayer with 95–100% confluency had been established.

### Blood sampling

Whole blood (WB) from different healthy volunteers was freshly drawn into Sarstedt Monovette^®^ tubes containing 0.106 mol/l sodium citrate (Sarstedt AG, Nümbrecht, Germany) and used for experiments within 60 min after blood sampling. The blood sampling was approved by the ethics committee of the canton of Bern and all volunteers gave informed consent.

### Clot formation experiments with rMASP-1

**Fig 2:**
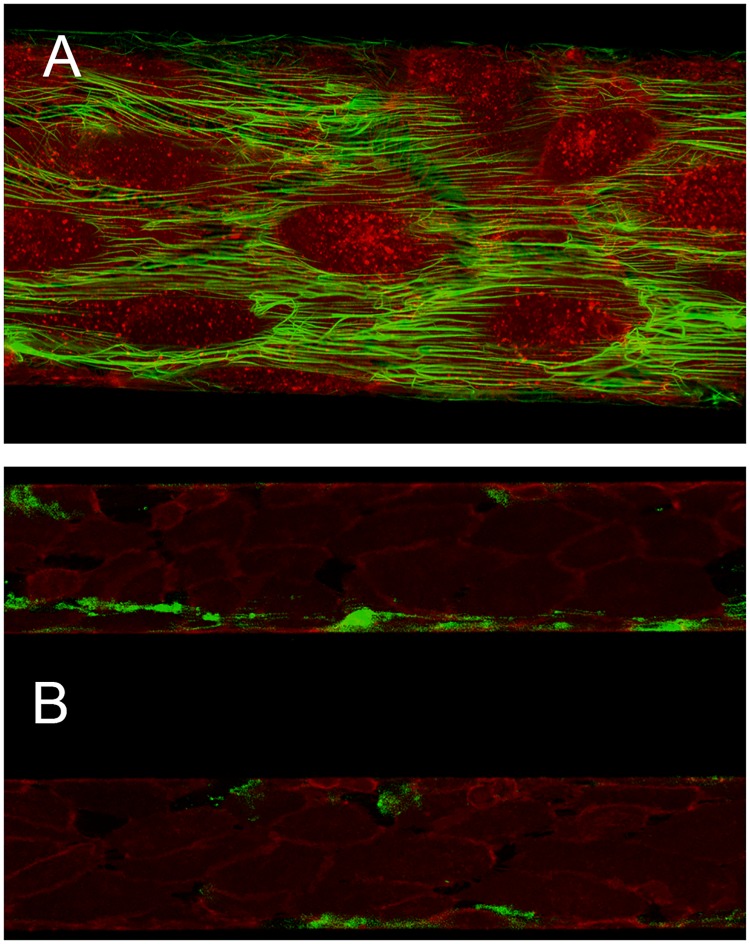
Exemplary microscopic images of the flow model. (A) Endothelial cells stained in red and green-labelled fibrin at 40x magnification. (B) Exemplary image taken at 10x magnification from a time course experiment, showing two parallel channels perfused with whole blood in presence (top channel) or absence (bottom channel) of zymosan at 18 min after recalcification. Endothelial cells are stained in red, fibrin in green.

The channels of the chip were perfused with EGM-2 containing CellMask^™^ staining (final concentration 1 μl/ml, excitation/emission wavelengths 554/567 nm; Life Technologies) for 10 min at a flow rate of 2 μl/min to stain the endothelial cell monolayer. The citrated WB was supplemented with prestained fibrinogen (final concentration 50 μg/ml, Alexa Fluor^®^ 488 conjugate, excitation/emission 495/519 nm; Molecular Probes, Eugene, Oregon, USA) and recalcified with CaCl_2_ (final concentration 12.5 mmol/l). An exemplary image of stained endothelial cells and fibrin deposition is shown in Fig 2A. Immediately after recalcification, the WB was supplemented with either rMASP-1cf (final concentration 10 μg/ml, 220 nmol/l) or SGMI-1 (20.4 μg/ml, 5 μmol/l) and channels were perfused with the blood samples at a flow rate of 2 μl/min and observed with confocal microscopy at 10x magnification (LSM 710 confocal microscope with Zen software Version 2.1.; Carl Zeiss AG, Oberkochen, Germany). The time from recalcification until the first appearance of immobilized fibrin in a channel was recorded. The deposition of fibrin was used as a measurement for clot formation. For comparisons between groups, IBM SPSS Statistics Software Version 24 was used.

### Complement activation and inhibition experiments

Freshly drawn citrated WB was supplemented with corn trypsin inhibitor (CTI, Haematologic Technologies, Essex Junction, Vermont, USA) at a final concentration of 2.86 μmol/l (40 μg/ml) to inhibit contact pathway activation during prolonged incubation of the blood. Subsequently, pre-activated zymosan (activation of zymosan by boiling in saline for 2 h, performed by the manufacturer according to Minta et al., 1983 [28], final concentration 0.5 mg/ml, CompTech, Tyler, Texas, USA), or the complement inhibitors rSALO (final concentration 5 μg/ml, 462 nmol/l) and/or SGMI-1 (final concentration 20.4 μg/ml, 5 μmol/l) were added to the WB samples and incubated on a rotator at 8 rpm for 15 min at 37°C. Subsequently, the WB samples were recalcified and immediately thereafter used to perfuse the channels for time course imaging (period of 18 min at cycles of 15 seconds) (Fig 2B). The images were evaluated with ImageJ (Version 1.51k, Wayne Rasband, National Institute of Mental Health, Bethesa, Maryland, USA) by measuring the fluorescence signal of immobilized fibrin within the channels over time. The results were displayed in graphs showing fluorescence intensity over time (SigmaPlot 13.0, Systat Software Inc., San Jose, CA, USA).

## Results

Recently, we demonstrated that MASP-1 can activate coagulation factors and induce prothrombin-dependent clot formation in isolated/purified systems [12,13]. The aim of the present study was to investigate the relevance of MASP-1 and complement activation to clot formation in a close-to-physiological environment. Here, we show for the first time in an endothelialized, microvascular whole blood flow system that the action of MASP-1 and complement activation enhance fibrin clot formation whereas their inhibition reduces or even almost abolishes fibrin deposition.

### rMASP-1cf accelerates clot formation in the microvascular flow model

**Fig 3:**
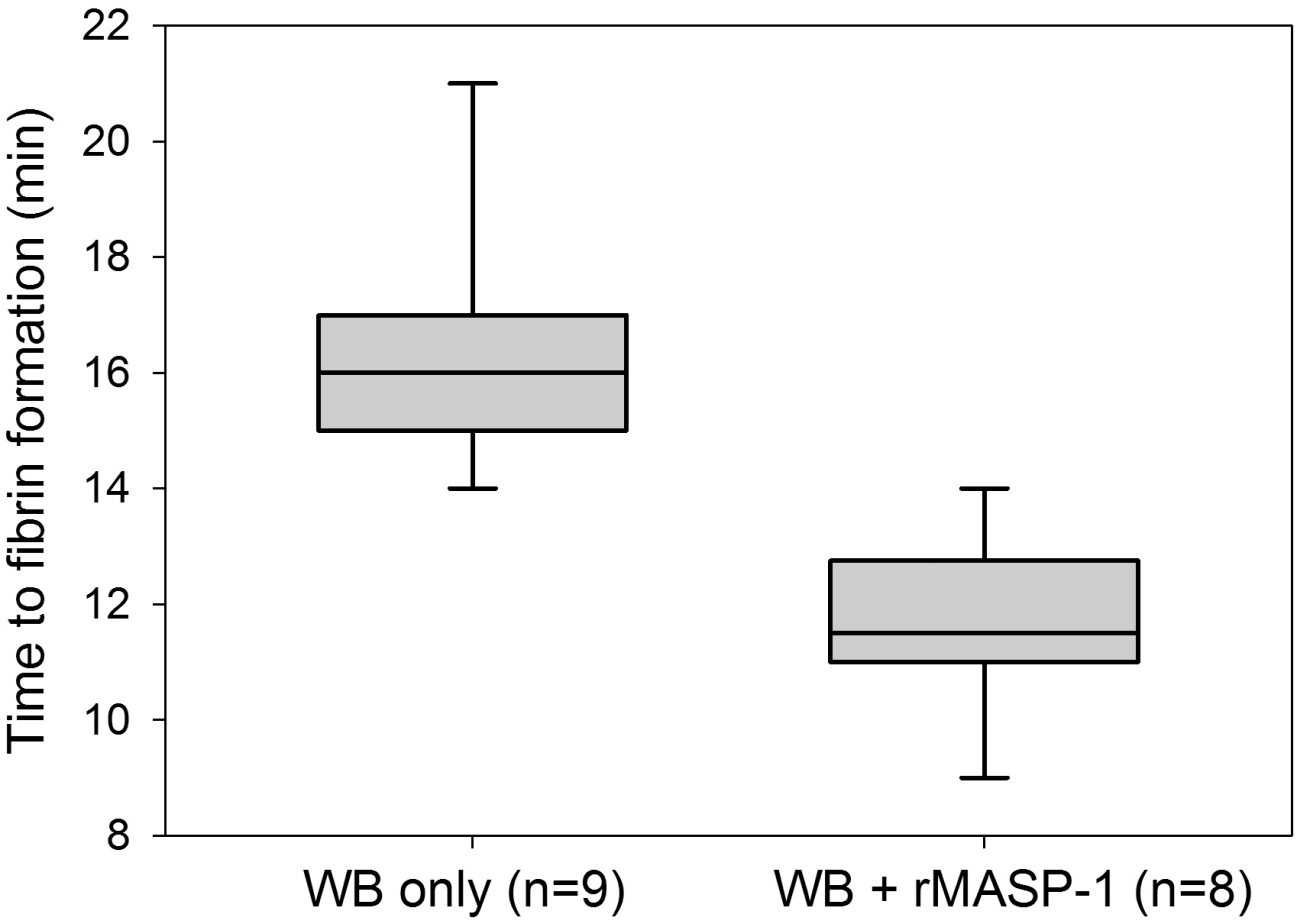
Effect of mannan-binding lectin-associated serine protease-1 recombinant catalytic fragment (rMASP-1cf) on clot formation in the microvascular whole blood flow model. The time from recalcification until the first appearance of immobilized fibrin in a channel was recorded to measure the effect of rMASP-1cf on clot formation. Box plots represent the 25–75% percentiles with the median shown as line. The whiskers represent the 10 and 90% percentiles.

Endothelialized microchannels were perfused with WB freshly drawn from healthy donors. Recalcified WB required an average of 16.3 min (SD 2.1 min, range 14–21 min, n = 9) to form the first immobilized fibrin clots. Subsequently, the experiments were repeated using recalcified WB samples supplemented with 10 μg/ml active rMASP-1cf. This resulted in an average time to first fibrin clots of 11.6 min (SD 1.5 min, range 9–14 min, n = 8). Thus, as shown in Fig 3, MASP-1 accelerated fibrin formation significantly compared with recalcified WB only (p<.001, Mann-Whitney test).

### Inhibition of endogenous MASP-1 by SGMI-1 delays clot formation

**Fig 4:**
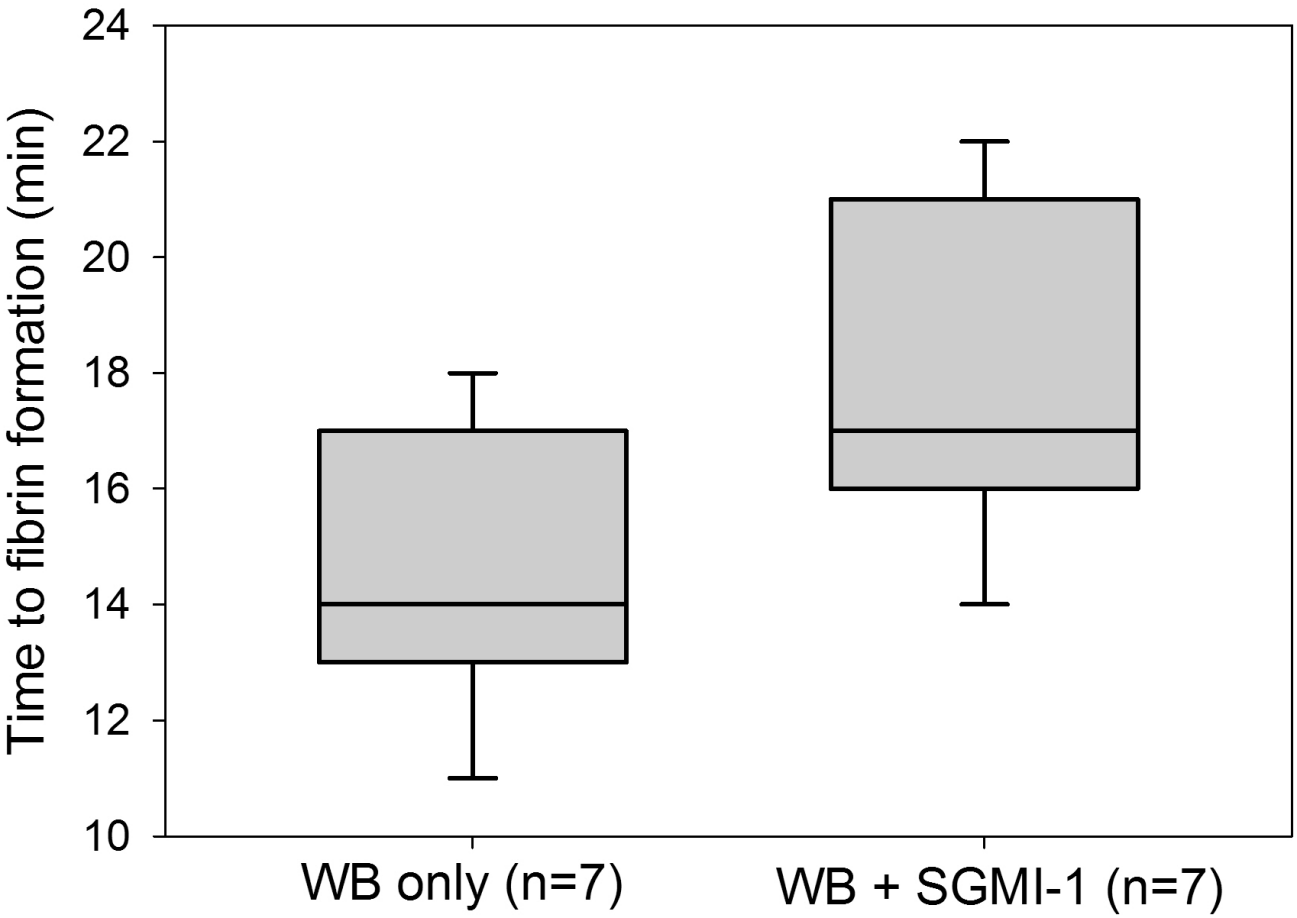
Effect of *Schistocerca gregaria* protease inhibitor (SGPI)-based MASP inhibitor-1 (SGMI-1) on clot formation in the microvascular whole blood flow model. The time from recalcification until the first appearance of immobilized fibrin in a channel was recorded to measure the effect of SGMI-1 on clot formation. Box plots represent the 25–75% percentiles with the median shown as line. The whiskers represent the 10 and 90% percentiles.

In view of the recently reported finding that MASP-1 and MASP-2 are activated in the course of clot formation [14], we wanted to evaluate to what extent endogenous MASP-1 (without addition of rMASP-1cf) would affect clot formation in the microvascular flow model. Therefore, microchannels were perfused with recalcified WB samples (n = 7) in the presence or absence of the MASP-1 inhibitor SGMI-1. WB in the absence of SGMI-1 showed an average fibrin clot formation time of 14.4 min (SD 2.44 min, range 11–18 min) while addition of SGMI-1 yielded an average clotting time of 17.9 min (SD 2.79 min, range 14–21 min). Thus, as shown in Fig 4, inhibition of endogenous MASP-1 by SGMI-1 was associated with a significant delay in the appearance of first immobilized fibrin (p<0.02, Wilcoxon signed rank test).

### Complement activation by zymosan increases clot formation

In a next step, we evaluated whether and in what manner activation of all three pathways of the complement system by zymosan would affect whole blood clot formation in the microvascular flow model. Five out of six samples (shown in S1 Fig) showed either earlier or more pronounced fibrin formation in WB with zymosan compared to clot formation in recalcified WB only. The variability observed in these experiments may—at least in part—be due to the individual response to zymosan. As most human serum contains natural antibodies to yeast resulting in classical pathway activation [29], varying amounts of anti-zymosan antibodies may result in a varying degree of complement activation. Other possible explanations include pre-activation of complement or coagulation in individual donors or samples, or even artefacts such as cellular particles occluding the channel. Overall, however, the results suggested that complement activation by zymosan increased fibrin clot formation in WB.

### Inhibition of the classical and/or lectin pathway reduces clot formation

**Fig 5:**
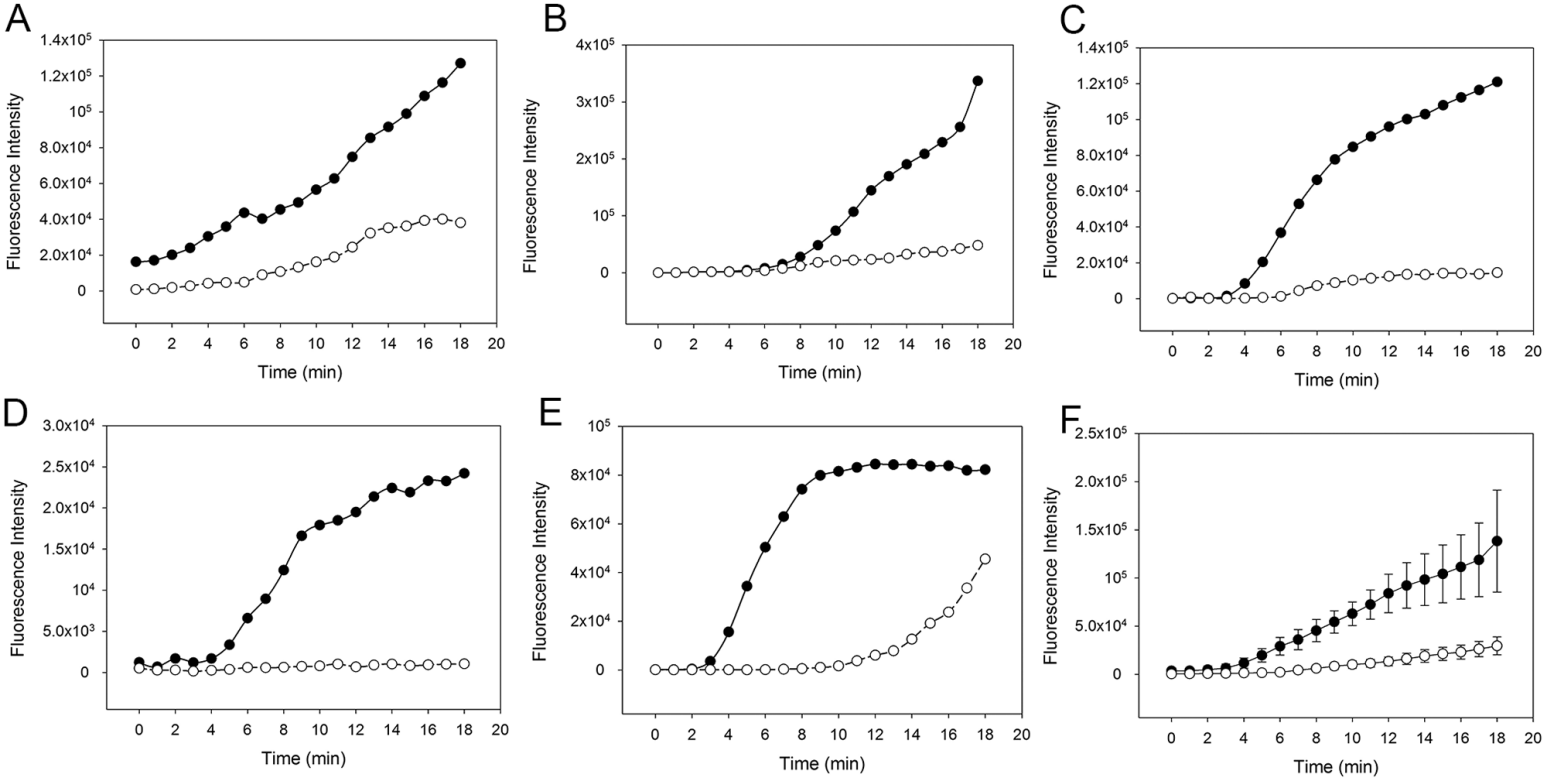
Effect of the classical pathway inhibitor recombinant salivary anti-complement from *L*. *longipalpis* (rSALO) on clot formation in whole blood (WB) with an activated complement system. The amount of fibrin forming over time is expressed as fluorescence intensity of green-fluorescent-labelled fibrinogen. Solid circles: Clot formation in zymosan-activated and recalcified WB without rSALO. Open circles: Clot formation in zymosan-activated and recalcified WB in the presence of rSALO. (A)-(E) Results from five individual experiments. (F) Average clot formation of (A)-(E) shown as mean (±SEM).

**Fig 6:**
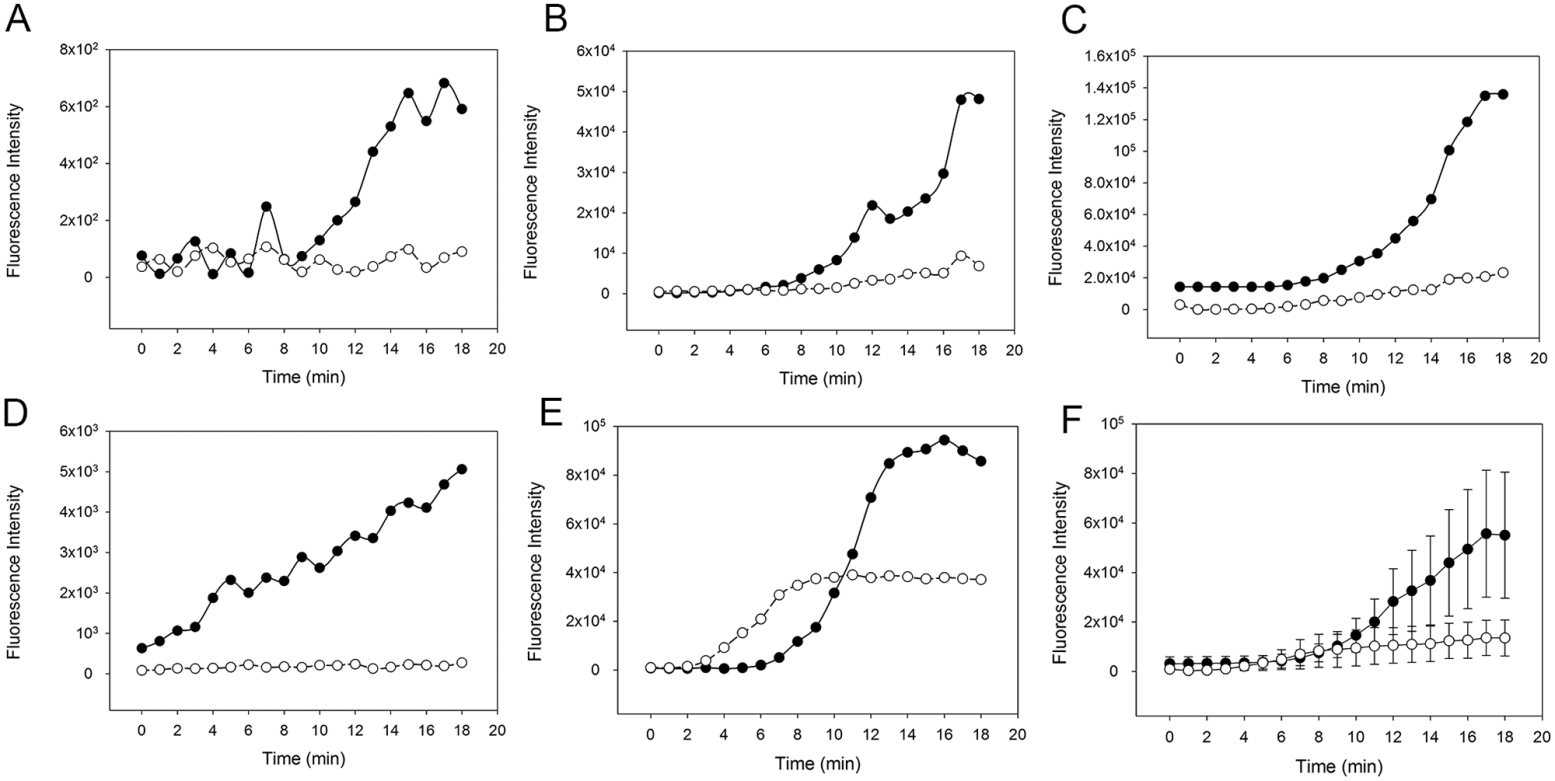
Effect of the complement inhibitors recombinant salivary anti-complement from *L*. *longipalpis* (rSALO, classical pathway) and *Schistocerca gregaria* protease inhibitor (SGPI)-based MASP inhibitor-1 (SGMI-1, lectin pathway) on clot formation in whole blood (WB) with an activated complement system. The amount of fibrin forming over time is expressed as fluorescence intensity of green-fluorescent-labelled fibrinogen. Solid circles: Clot formation in zymosan-activated WB supplemented with rSALO only. Open circles: Clot formation in zymosan-activated WB supplemented with rSALO and SGMI-1. (A)-(E) Results from five individual experiments. (F) Average clot formation of (A)-(E) shown as mean (±SEM).

In order to assess the individual contributions of the classical (CP) and lectin (LP) pathways, we next investigated fibrin clot formation in WB with zymosan-activated complement in presence or absence of the CP inhibitor rSALO. Inhibition of the CP by rSALO showed a delayed and reduced clot formation suggesting that the CP contributes to clot formation (Fig 5). Addition of both MASP-1 inhibitor SGMI-1 and rSALO to zymosan-activated WB did further decrease and in some samples almost abolish clot formation (Fig 6), suggesting that the LP does contribute to clot formation albeit the effect may be weaker compared with the CP. In summary, these results showed that inhibition of CP and LP in zymosan-activated WB reduced fibrin clot formation in our microvascular whole blood flow model.

## Discussion

In recent years the crosstalk between the complement and the coagulation systems has moved into the focus of interest. With more and more interactions being discovered, an overall picture emerges of two tightly interwoven, related systems standing in a firmly regulated balance. We and others have demonstrated that the lectin pathway protease MASP-1 affects the coagulation system on various levels and promotes clot formation. However, many experiments on the effects of MASP-1 on coagulation were conducted in isolated and static systems such as plasma or with purified proteins in order to elucidate the underlying mechanisms. Here we aimed at evaluating the role and relevance of MASP-1 and complement activation in clot formation in a microvascular whole blood flow model which provides the closest model to human physiology and thus the best human *in vitro* system. The parallel channels featured by this model allow to simultaneously observe two blood samples under the same conditions, making it an excellent tool to evaluate the effects of different activators and inhibitors on clot formation. This setting also helps to reduce the impact of the inter- and intra-individual variability that is expected in a complex experimental environment with numerous influencing factors, including the current physiological state and genetic predisposition of the individual.

For the first time we demonstrate in a microvascular flow model close to human physiology, that addition of active rMASP-1cf significantly accelerates fibrin clot formation in WB and that addition of the MASP-1 inhibitor SGMI-1 delays clot formation. In standard coagulation assays (TT, PT, aPTT), SGMI-1 did not show any effects [26] which may be due to the assay conditions. TT, PT and aPTT are accelerated by using coagulation activators which may not leave enough time for endogenous MASP-1 activation and action.

The observed effects of rMASP-1cf on the coagulation are not surprising as MASP-1 has been shown to directly activate prothrombin and FXIII and to interact with fibrinogen/fibrin, platelets and endothelial cells [9,11–14,30]. Furthermore it is also known that downstream complement molecules, such as MASP-2, C3a, C5a and the C5b-9, affect the coagulation system, suggesting that MASP-1 has also indirect effects on coagulation via complement activation [31–33]. C3a, C5a and C5b-9 are part of the common complement pathway and can be generated independently from the lectin pathway by the classical and alternative pathway [4]. Indeed it has been shown that the CP and the AP also affect coagulation [1–3]. This is in accordance with our observation that inhibition of the CP by rSALO diminished clot formation in the microvascular flow model.

Our results show that both LP and CP simultaneously affect coagulation as inhibition of both pathways by SGMI-1 and rSALO almost suppresses clot formation. It is notable that inhibition of the CP seems to have a stronger effect on the fibrin formation than inhibition of the LP by SGMI-1-conferred MASP-1 blockage in zymosan-activated WB. It may point towards a weaker effect of the LP on coagulation which is masked by the influence of the CP in an environment where all complement pathways are active. The observed suppression of clot formation in presence of SGMI-1 and rSALO in zymosan supplemented blood also suggests that zymosan itself does not significantly interact with the coagulation cascade in a direct manner. Although it cannot be absolutely excluded as there still remains clot formation on a low level, we assume that a direct effect of zymosan on the coagulation is negligible in this setting. Further, one should also take into consideration that in the above assay zymogen-activated WB was supplemented with the FXIIa inhibitor CTI to prevent contact pathway activation. While in the context of recalcified WB with an intact contact pathway (without CTI) MASP-1 significantly contributed to clot formation, in the context of contact pathway inhibited WB this contribution is diminished. This might suggest that MASP-1 exerts its procoagulant effect at least in part through enhancing the contact pathway, and this contribution cannot manifest when the contact pathway is blocked by CTI.

Although we consider the microvascular whole blood flow model closest to human physiology and the best human *in vitro* system in regard to the presence of endothelial and blood cells, a vessel structure and flow conditions, there are still limitations to be taken into account. So far, the sample handling time and setup of the system did not allow the use of native, uncitrated blood, which would be preferable as citrated/recalcified blood may have a tendency for hypercoagulation [34]. A critical point is the cell confluency within the channels. Endothelial cells in an incomplete monolayer may not exhibit their full anticoagulant properties, and subendothelial structures may activate coagulation. It can be difficult to assess if channels are 100% confluent or not, as not every part of the device (especially the inlet area) can be thoroughly examined for uncovered patches. Yet, as both channels are seeded with the same number of endothelial cells from the same batch and are treated in the same way, we have rarely seen a profound difference in confluency in our hands and we therefore judge the results obtained in parallel channels as very comparable.

## Conclusions

In conclusion, our work underpins the role of MASP-1 as an important link between the complement and coagulation systems. We show for the first time in a microvascular whole blood flow model that MASP-1 inhibition and especially inhibition of both LP and CP significantly diminishes fibrin deposition upon complement activation. In healthy individuals, the complement and coagulation systems are tightly regulated and pro-/anti-inflammatory and pro-/anti-coagulant effects are well balanced. Under pathological conditions, e.g. sepsis, or atherosclerosis and diabetes, where this balance is disturbed, the interactions between the two systems may promote the development of thrombotic complications. Complement activation in general and the LP and MASP-1 in particular may represent promising targets for novel preventive and therapeutic strategies. Further research into complement-coagulation interactions is clearly needed and the microvascular flow model can serve as a powerful tool in this field.

## Supporting information

S1 Fig Effect of zymosan-activated complement on clot formation. The amount of fibrin forming over time is expressed as fluorescence intensity of green-fluorescent-labelled fibrinogen. Solid circles: Clot formation in recalcified whole blood with zymosan. Open circles: Clot formation in recalcified whole blood without zymosan. (A)-(F) Results from six individual experiments.
(TIFF)
