# Supplementary figures and images for: MASP-1 of the complement system enhances clot formation in a microvascular whole blood flow model
Source: PLoS One. 2018 Jan 11;13(1):e0191292. doi: 10.1371/journal.pone.0191292 (PMC5764403; doi:10.1371/journal.pone.0191292)

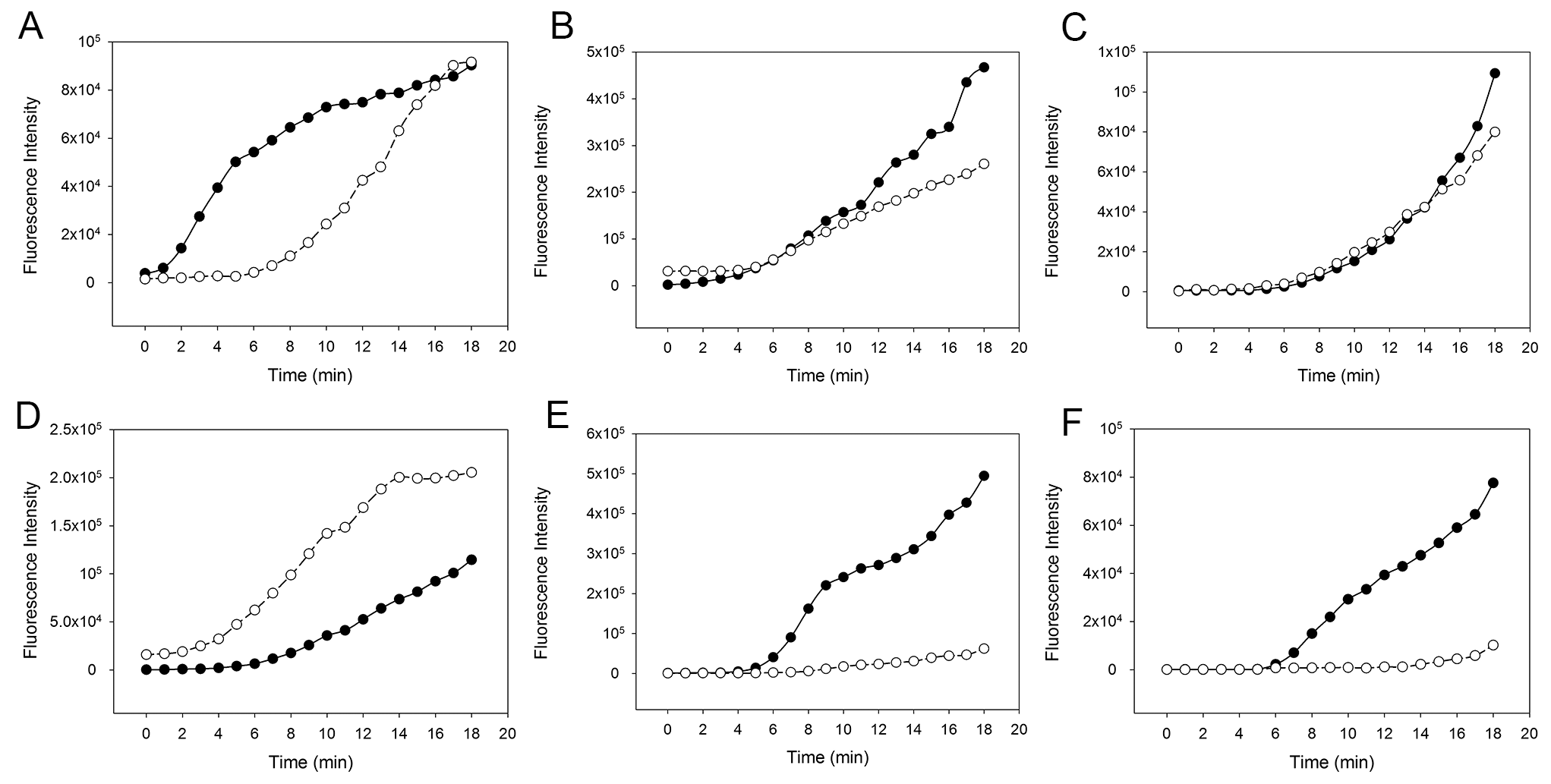

Supplement: S1 Fig — The amount of fibrin forming over time is expressed as fluorescence intensity of green-fluorescent-labelled fibrinogen. Solid circles: Clot formation in recalcified whole blood with zymosan. Open circles: Clot formation in recalcified whole blood without zymosan. (A)-(F) Results from six individual experiments. (TIFF) [file pone.0191292.s001.TIFF]
